# Association between doctor-patient familiarity and patient-centred care during general practitioner's consultations: a direct observational study in Chinese primary care practice

**DOI:** 10.1186/s12875-021-01446-4

**Published:** 2021-05-28

**Authors:** Chenwen Zhong, Mengping Zhou, Zhuojun Luo, Cuiying Liang, Lina Li, Li Kuang

**Affiliations:** 1 Department of Health Policy and Management, School of Public Health, Sun Yat-Sen University, Guangzhou, 510080 Guangdong China; 2 Jockey Club School of Public Health and Primary Care, The Chinese University of Hong Kong, Hong Kong, 999077 China

**Keywords:** Doctor-patient familiarity, Davis Observation code, Consultation, Patient-centred care, Primary care

## Abstract

**Background:**

Patient-centred care is a core attribute of primary care. Not much is known about the relationship between patient-centred care and doctor-patient familiarity. This study aimed to explore the association between general practitioner (GP) perceived doctor-patient familiarity and the provision of patient-centred care during GP consultations.

**Methods:**

This is a direct observational study conducted in eight community health centres in China. Level of familiarity was rated by GPs using a dichotomized variable (Yes/No). The provision of patient-centred care during GP consultations was measured by coding audiotapes using a modified Davis Observation Code (DOC) interactional instrument. Eight individual codes in the modified DOC were selected for measuring the provision of patient-centred care, including ‘family information’, ‘treatment effects’, ‘nutrition guidance’, ‘exercise guidance’, ‘health knowledge’, ‘patient question’, ‘chatting’, and ‘counseling’. Multivariate analyses of covariance were adopted to evaluate the association between GP perceived doctor-patient familiarity and patient-centred care.

**Results:**

A total of 445 audiotaped consultations were collected, with 243 in the familiar group and 202 in the unfamiliar group. No significant difference was detected in overall patient-centred care between the two groups. For components of patient-centred care, the number of intervals (1.36 vs 0.88, *p* = 0.026) and time length (7.26 vs. 4.40 s, *p* = 0.030) that GPs spent in ‘health knowledge’, as well as time length (13.0 vs. 8.34 s, *p* = 0.019) spent in ‘patient question’ were significantly higher in unfamiliar group. The percentage of ‘chatting’ (11.9% vs. 7.34%, *p* = 0.012) was significantly higher in the familiar group.

**Conclusions:**

This study suggested that GP perceived doctor-patient familiarity may not be associated with GPs’ provision of patient-centred care during consultations in the context of China. Not unexpectedly, patients would show more health knowledge and ask more questions when GPs were not familiar with them. Further research is needed to confirm and expand on these findings.

## Background

Patient-centred care is a core value of general practice and is often connected to high quality of patient care, with a definition of providing care responsive to patient preferences, needs, and values and ensuring that patient values guide all clinical decisions [1, 2]. It indicates that patients’ values and preferences are becoming the guidance of all aspects of health care for supporting their realistic health and life goals [3]. Patient-centred care is always achieved through a dynamic relationship among individuals and implemented during doctor-patient consultations. It consists of six domains: fostering healing relationships between patients and doctors, exchanging information, responding to emotions, managing uncertainty, practising shared decision-making, and enabling patient self-management [4]. Patient-centred care may increase patient satisfaction and empowerment, as well as reduce symptom severity, rate of referral, and health costs [5, 6].

Another important concept for general practice is doctor-patient familiarity [7]. Doctor-patient familiarity refers to the familiar level between general practitioners (GPs) and patients. Assessment of doctor-patient familiarity could be conducted from both GP and patient perspectives. Current measures for doctor-patient familiarity are usually subjective. For example, to assess patient perceived familiarity with GP, patients may be asked to rate whether they are familiar with the GP in most studies, while for GP perceived familiarity with patients, it is measured by asking GPs whether they are familiar with the patient [8–10]. Shers’s study showed that GP’s familiarity with patients improves patients’ experience of primary care and personal continuity [8]. Patients may see a familiar GP to a high extent, regardless of reasons for visits, and perceived seriousness of symptoms [8]. Jabaaij’s study indicated that when GPs were familiar with the patient, GPs may discuss more and provide more information to the patient towards the topic in questions [9]. Yu’s study analyzed the consultation length and waiting time of general practice in community health centres in China and found that the consultation length was significantly related to doctor-patient familiarity [10]. Higher levels of doctor-patient familiarity were associated with increased patient satisfaction, better preventive care, patient education, and also fewer emergency department visits [11–13].

In China, the relationship between patients and GPs are being strengthened according to the policy direction set by the Chinese government and the Guidance on the Promotion of Family Practice Contract Services issued by the National Health Commission in 2016. It is anticipated that fostering a closer relationship would have a substantial impact on the content of healthcare delivered by GPs. For example, the doctor-patient familiarity level may affect GPs’ provision of patient-centred care [14]. However, not much is known about this issue. Hence, this study was conducted to explore the association between GP perceived doctor-patient familiarity and the provision of patient-centred care. The study findings may inform the effectiveness of implementing family practice contract services. On the other hand, after analyzing the characteristics of GP consultations, it may point out ways on how to enhance the attributes of patient-centred care during GP consultations, thereby improving patients’ experience of primary care and also their prognostic outcomes ultimately.

GP consultation is the core activity of general practice, and the most important approach to implement patient-centred care. In this study we assessed the provision of patient-centred care during GP consultations based on a direct observational study of primary care and using Davis Observation Code, an instrument used to characterize doctor-patient interaction during GP consultations. The main purpose of this study is to investigate the relationship between GP perceived doctor-patient familiarity and the provision of patient-centred care in the consultations.

## Methods

### Study design

This study was part of a direct observational study of primary care that provided a profile of GP consultations in community health centres (CHCs) in China [15]. It was conducted between July 2018 and January 2019. Detailed methodological section regarding GPs and patient’s recruitment, sample size, data collection, and indicators justification have been described in our previously published paper [15]. Briefly, we recruited subjects from eight CHCs in Guangzhou and Shenzhen city, China. We adopted a nested sampling method and recruited a total of 17 eligible GPs and 445 patients. Principle investigator (LK) of this project firstly contacted the CHCs and invited GPs to participate in this study. GPs were recruited in a voluntary manner. Inclusion criteria of GPs included rich clinical experience (defined as ‘over 10-year clinical experience), a large volume of outpatient visits per day (defined as ‘more than 40 outpatient visits per day’), and signed informed consent to participate in the study. After the recruitment of GPs, all patients coming to see the GPs on the observation day were considered as potential subjects for this study. Patients included in this study should be older than 18, and orally agree to participate in this study.

### Data collection

We conducted this direct observational study in GP’s consultation room. During the observation period, two observers sat at a corner of GP’s office, asked for patients’ informed consent before the consultation process, and collected data of GP consultation using audiotape and a pre-designed direct observation form for each patient. The direct observation form collected additional data of doctor-patient interaction that cannot be collected by audiotapes during the silence time, e.g., patients’ reasons for visit, with or without a company when seeing a GP, silence interaction between GPs and patients such as medical record reading and prescription on the computer, and also the familiarity between GP and the patient. Start time and end time were recorded for each type of interactions. After consultation, patients were invited to fill in a survey to collect their socioeconomic characteristics, health service utilization, and other relevant information. A small incentive gift (such as toothpaste or tissues) was sent to patients who participated in this study.

To reduce the possibility of the Hawthorne effect, we adopted several strategies as follows. Firstly, GPs and patients were asked to act as normal as the observers were not aimed to do any assessments or judgements. Secondly, observers sat at the most unattractive place of the room and avoided eye contact with GPs and patients. Thirdly, observers were asked to dress in a white coat since the presence of a medical student is a normal occurrence during many outpatient visits to patients and GPs.

### Data analysis

#### Measures for patient-centred care

We used a modified Davis Observation Code (DOC) to characterize the patient-centred care practice style [16]. DOC was an important interactional analysis instrument that has been validated and frequently used to detect physician practice style differences in a variety of studies [17–21]. Bertakis and his colleagues further developed the modified DOC patient-centred care interactional analysis system [16, 20, 22] by incorporating the five key dimensions of patient-centred care proposed by Mead and Bower: understanding the patient’s illness within a broader biopsychosocial context, appreciating the patient’s experience of illness, advocating for an egalitarian relationship, creating a therapeutic alliance, and acknowledging the impact of the participants’ personal qualities on the medical encounter [1]. Based on Bertakis’s theory, a total of eight individual codes of the modified DOC were included to measure patient-centred care, including ‘family information’, ‘treatment effects’, ‘nutrition guidance’, ‘exercise guidance’, ‘health knowledge’, ‘patient question’, ‘chatting’, and ‘counseling’.

#### Data coding

We used NVIVO software to divide the whole visit time into every 15-second (15-s) time interval. Four coders recognized each DOC code in the 15-s time intervals and recorded the occurrence and duration time of each DOC code accordingly based on the audio-record and direct observation form. Several DOC codes may occur in the same time interval. Before coding, we trained the coders with several pieces of audiotapes iteratively until a consensus was made on the operational definition of each code. Through the coding process, regular meetings were held every week to resolve any uncertainties. Krippendorff’s alpha [23] was adopted to determine the inter-rater reliability among coders, which showed a good result with a coefficient of 0.81 in this study.

#### Indicators

We calculate the detailed duration time spent in each visit and each component. For patient-centred care, the number of intervals in which the eight components mentioned above occurred was recorded, respectively. We also expressed the number of intervals for each code as a percentage. The percentage may indicate GPs’ priority in the consultations. The time spent in patient-centred care was calculated by summing up the duration of the eight codes.

#### Measures for GP perceived doctor-patient familiarity

GP perceived doctor-patient familiarity was collected after each consultation. The variable, ‘knowing the patient well’ answered by GP, with binary results ‘Yes’ or ‘No’ was used as a proxy measure for GP perceived doctor-patient familiarity [9]. When the GP rates “yes”, it indicates that he knows the patient well. In this case, the GP may interact with the patient regularly and have comprehensive knowledge of the patient. A close relationship may have been fostered between the GP and patient before the current consultation.

#### Statistical analysis

We performed chi-square analyses to compare patient’s socio-demographic characteristics, health status, and health utilization between patients that GPs were familiar with or not. We used multivariate analyses of covariances (ANCOVAs) to compare the mean interval number, percentage, and time length of patient-centred care between the two groups of patients that GPs were familiar with or not after adjusted by patient sex, age, marital status, education, monthly household income, employment status, household status, health insurance, health status, reasons for visit, number of visits to the CHC in the last year, whether have a family doctor or not and with or without chronic diseases. A *P* value of less than 0.05 was considered statistically significant. All analyses were conducted using IBM SPSS 26.

## Results

### Patient characteristics

A total of 445 consultations were collected, with 243 in the familiar group and 202 in the unfamiliar group. Table 1 presents the social demographic characteristics, health status, health utilization between the two groups of patients. The majority age range in the familiar group was older than 65 years old (49.8%), while in the unfamiliar group, it was 45–65 with a percentage of 44.6%. In both groups, most respondents were female, married, unemployed, monthly household income less than 5000 CNY (nearly 764.6 USD), local residence, and with Urban Employee Basic Medical Insurance. For health status, half of the patients in both groups reported ‘general’. Among patients in the familiar group, 87.7% were with chronic diseases, 97.9% visited the CHC more than three times last year, and 68.6% reported having a family doctor. However, for patients in the unfamiliar group, 6.8% had no health insurance covered, and 67.3% reported no family doctor. There were significant differences in age, marital status, education, employment status, monthly household income, household status, social medical insurance, health status, chronic disease condition, number of CHC visits in the last year, and whether they had a family doctor between the two groups.

**Table 1:** Patients characteristics, health status, health utilization and patient visits’ characteristics between the two groups

	Familiar (*N* = 243)		Unfamiliar (*N* = 202)		*p*
	n	%	n	%	
Sex					0.185
Male	92	37.9	89	44.1	
Female	151	62.1	113	55.9	
Age(years)					< 0.001
< 45	11	4.5	65	32.2	
45–65	111	45.7	90	44.6	
> 65	121	49.8	47	23.3	
Marital status					< 0.001
Unmarried	0	0	21	10.4	
Married	201	82.7	171	84.7	
Divorced or widowed	42	17.3	10	5.0	
Education					< 0.001
Primary school or below	121	49.81	80	39.6	
Middle/high school	95	39.1	69	26.2	
Bachelor’s degree or above	27	11.1	53	26.2	
Employment status					< 0.001
Employed	21	8.6	85	42.1	
Retired	207	85.2	98	48.5	
Unemployed	15	6.2	19	9.4	
Monthly household Income (CNY)					< 0.001
< 5000	199	81.9	125	61.9	
5000–10,000	32	13.2	46	22.8	
> 10,000	12	4.9	31	15.3	
Household status					< 0.001
Local	225	92.6	136	67.3	
Non-Local, residence time > 6 months	18	7.4	59	29.2	
Non-Local, residence time < 6 months	0	0	7	3.5	
Social medical insurance (*N* = 432)					< 0.001
Urban Employee Basic Medical Insurance	189	78.4	106	55.5	
Urban Residence Basic Medical Insurance	38	15.8	19	9.9	
Basic Medical Insurance	9	3.7	49	25.7	
Others (new rural cooperative medical system)	5	2.1	4	2.1	
Without Medical Insurance	0	0	13	6.8	
Health status					0.024
Poor	34	14.0	13	6.4	
General	134	55.4	114	56.4	
Good	74	30.6	75	37.1	
Chronic diseases					< 0.001
Yes	213	87.7	116	57.4	
No	30	12.3	86	42.6	
Number of visits in the CHC in last year					< 0.001
1–2	5	2.1	56	27.7	
≥ 3	238	97.9	146	72.3	
Having a family doctor					< 0.001
Yes	164	68.6	55	32.7	
No	75	31.4	113	67.3	
Reasons for visits					< 0.001
Acute illness	73	30.0	115	56.9	
Chronic disease: follow up or refill a prescription	145	59.7	67	33.2	
Chronic disease: flare up	4	1.6	5	2.5	
Counseling for well care or disease care	20	8.2	10	5.0	
Others (health examination, rehabilitation, etc	1	0.4	5	2.5	

### Visit Characteristics

Patients’ reasons for the current visit were significantly different between the two groups of patients that GPs were familiar with or not. In familiar groups, 59.7% of patients stated their reason for the current visit was for the follow-up of their chronic disease, while 56.9% of patients in the unfamiliar group reported their reason for the current visit was for acute illness (Table 1). The average time duration of visits in the familiar group and the unfamiliar group were 5.81 and 5.08 min, respectively. As for GP’s work density, at any 15-s interval during one visit, approximately 1.84 and 1.89 DOC codes were occurring simultaneously in the two groups, respectively (Table 2). We observed no relevant differences in the duration of visits and GP’s work density between the two groups of patients that GPs were familiar with or not, controlling for patient sex, age, marital status, education, monthly household income, employment status, household status, health insurance, health status, reasons for visit, number of visits to the CHC in the last year, whether have a family doctor or not and with or without chronic diseases.

**Table 2:** Visit Characteristics between the two groups

	Familiar (*N* = 243)		Unfamiliar (*N* = 202)		Diff^a^ (I-II)	*p*-diff
	Mean (I)	SE	Mean (II)	SE		
Duration of visits (in minutes)	5.81	0.30	5.08	0.24	-0.722	0.084
Number of components appeared in each interval	1.84	0.06	1.89	0.08	-0.044	0.675

Keys: Values are adjusted for patient sex, age, marital status, education, monthly household income, employment status, household status, health insurance, health status, reason for visit, number of visits to the CHC in the last year, whether have a family doctor or not, with or without chronic diseases. SE: standard error

Diff^a^: absolute difference between familiar group and unfamiliar group

### GP perceived doctor-patient familiarity and the provision of patient-centred care

Table 3 shows the associations between patient-centred care and level of familiarity after adjusting for patient sex, age, marital status, education, monthly household income, employment status, household status, health insurance, health status, reasons for visit, number of visits to the CHC in the last year, whether have a family doctor or not, and with or without chronic diseases. In general, seeing through the three indicators, there were no significant differences in patient-centred care during GP consultations between the familiar and unfamiliar groups. For the eight codes in patient-centred care, the results showed that the mean difference in the number of intervals in which ‘health knowledge’ occurred between the two groups was -0.484 (familiar vs. unfamiliar, *p* = 0.026). The detailed time length GPs spent in ‘health knowledge’ was also found to be marginally longer in the unfamiliar group than in the familiar group (7.26 s vs. 4.40 s, *p* = 0.030). Furthermore, the detailed time length that GPs spent in ‘patient questions’ was found to be longer in the unfamiliar group than in the familiar group (13.0 s vs. 8.34 s). On the other hand, we found that ‘chatting’ accounted for a larger percentage during GP consultations in the familiar group compared with the unfamiliar group (11.9 vs. 7.34, *p* = 0.012).

**Table 3:** Time spent in overall patient-centred care and each code during GP's consultations of the two groups

Codes	Interval number for each code in one visit, Mean ± SE				Percentage for each code in one visit (%), Mean ± SE				Time spent in each code in one visit (second), Mean ± SE			
	Familiar (I)	Unfamiliar (II)	Diff (I-II)	p^*^	Familiar (I)	Unfamiliar (II)	Diff (I-II)	P^*^	Familiar (I)	Unfamiliar (II)	Diff (I-II)	p^*^
Patient centred care	6.68 ± 0.66	7.03 ± 0.81	-0.346	0.759	33.0 ± 2.60	29.4 ± 3.20	3.624	0.418	43.4 ± 3.52	45.0 ± 4.33	-1.562	0.797
Family information	0.02 ± 0.01	0.01 ± 0.01	0.012	0.538	0.10 ± 0.05	0.02 ± 0.06	0.079	0.358	0.19 ± 0.12	0.11 ± 0.15	0.079	0.707
Treatment effect	1.37 ± 0.30	1.30 ± 0.37	0.066	0.665	2.73 ± 0.35	2.18 ± 0.43	0.549	0.360	3.37 ± 0.49	2.68 ± 0.61	0.688	0.416
Nutrition advice	0.44 ± 0.08	0.24 ± 0.10	0.197	0.168	1.77 ± 0.35	1.06 ± 0.43	0.709	0.242	3.81 ± 0.79	1.83 ± 0.97	1.981	0.145
Exercise advice	0.18 ± 0.45	0.09 ± 0.06	0.095	0.227	0.61 ± 0.19	0.52 ± 0.23	0.093	0.771	1.73 ± 0.46	0.46 ± 0.57	1.270	0.112
Health knowledge	0.88 ± 0.13	1.36 ± 0.16	-0.484	0.026*	4.30 ± 0.57	6.21 ± 0.71	-1.905	0.055	4.40 ± 0.77	7.26 ± 0.94	-2.862	0.030*
Patient question	1.46 ± 0.18	2.00 ± 0.23	-0.546	0.084	7.51 ± 0.80	8.09 ± 0.99	-0.577	0.677	8.34 ± 1.16	13.0 ± 1.42	-4.689	0.019*
Chatting	2.26 ± 0.27	1.95 ± 0.33	0.306	0.506	11.9 ± 1.05	7.34 ± 1.29	4.567	0.012*	19.4 ± 2.67	17.3 ± 3.29	2.050	0.656
Counselling	0.08 ± 0.04	0.07 ± 0.05	0.009	0.898	0.21 ± 0.10	0.23 ± 0.13	-0.023	0.897	0.84 ± 0.41	0.51 ± 0.50	0.323	0.646

Note: *SE* standard error. *Diff* absolute difference between familiar group and unfamiliar group

^*^ Multivariate analyses of covariance controlling for patient sex, age, marital status, education, monthly household income, employment status, household status, health insurance, health status, reason for visit, number of visits to the CHC in the last year, whether have a family doctor or not, with or without chronic diseases

## Discussion

### Summary

In this study, we compared patient-centred care during GP consultations using a modified DOC interactional analysis system between two groups of patients that GPs were familiars with or not. No significance was detected in the average time duration of visits and GPs’ work density per interval between the familiar group and the unfamiliar group. The interval number, percentage, and detailed time length for patient-centred care were not significantly different between the two groups, either. Seeing through the eight components of patient-centred care, the number of intervals, and detailed time length that GPs spent in the ‘health knowledge’ in the unfamiliar group were significantly higher than the familiar group. The detailed time length spent in the ‘patient question’ was also significantly longer in the unfamiliar group. However, the percentage of ‘chatting’ in the familiar group was significantly higher than in the unfamiliar group.

### Strength and limitations

To the best of our knowledge, this is the first study to report on the potential association of GP perceived doctor-patient familiarity on patient-centred care during GP consultations. The strengths of this study include adopting a modified DOC interactional analysis system to measure patient-centred care, using indicators of the number of intervals and percentage as well as accurate time length. There are several limitations to this study. Firstly, as with any observational study, no causal effect can be evaluated. Secondly, the level of GP perceived doctor-patient familiarity was measured by asking GPs whether they were familiar with the patients encountered, but not from both GP and patient perspective. Such measurement is subjective and may need further consideration. Future study may consider developing comprehensive scales for the measurement of doctor-patient familiarity in primary care settings. Thirdly, this study was conducted in the community health centres with well-developed general practice in China, thereby any generalization should be taken with caution. Finally, the number of consultations of each GP between the two groups was not distributed equally, which may cause some bias when comparing the association between familiarity and patient-centred care.

### Comparison with existing literature

Overall, we can see that GPs provided the same level of patient-centred care during GP consultations no matter whether GPs indicated they were familiar with the patient or not. According to Healthy People 2020, to achieve health equity, it is necessary to value all people equally with focused and ongoing efforts and avoid inequalities, historical and contemporary injustices, and eliminate health and health care disparities [24]. Therefore, results in this study may imply equity in the provision of patient-centred care during GP consultations regardless of familiarity between GPs and patients. To some extent, the results were similar to Jabaaij’s study that familiarity did not influence the GPs’ interaction with patients during the consultations [9]. GPs’ communication skills enable them to discuss any issue with patients tailoring with patients’ values and needs, thereby guaranteeing 'patient-centred care', whether they are familiar with the patient or not [25].

On the other hand, reasons for the nonsignificance findings may include the followings. Firstly, as the sample size of this study (445 visits, 243 visits in familiar group, and 202 in unfamiliar group) was calculated based on the whole project’s study design, in this study it may miss some statistically significant results due to limited statistical power. Future studies would be required to confirm the findings of this study. Secondly, the heavy workload of GPs in community health centres in China may result in a short consultation that focuses more on the medication prescription and history taking. Hence, GPs may spend less time and energy on patient-centred care. Thirdly, when a closer relationship was established between GPs and patients, patients may wish to reduce GPs workload by asking only questions related to their goals for visits and shortening the consultation time as possible. To some extent, the relationship between GP perceived doctor-patient familiarity and patient-centred care may hardly be detected through the quantitative measurement tool in this study. Future studies may consider evaluating the association between familiarity and patient-centred care by other approaches, such as qualitative interviews or mix-method studies. Lastly, in the practice of primary care in China, it can often be seen that most patients passively accept comments from the GPs, and rarely involved in the decision-making process for their disease management.

We also noticed that the number of intervals and detailed time length that GPs spent in ‘health knowledge’ and ‘patient questions’ in the unfamiliar group were significantly higher than the familiar group. The results indicated that more time is spent in patients spontaneously offering information about what they know or believe about health and disease during consultations in unfamiliar groups, compared with the familiar group. This is in contrast with a large cross-sectional study with 25,994 participants, which showed that knowing the doctor well resulted in considerably increased patient’s enablement [26]. On the other hand, patients that GPs were not familiar with were more active in the consultation. More time was spent in patients asking questions about diagnosis, treatment, side effects, history, or disease in the unfamiliar group. There may be some connection with Broom’s study that GP’s familiarity with the patient can lead to diminished medical vigilance, despite the manifest benefit on continuity [27].

In addition, we demonstrated a statistically significant association between GP perceived doctor-patient familiarity and the ‘chatting’ components during GP consultations. In this case, GPs and patients may spend more time discussing topics not related to the current visit such as small talk or humor which might be used to build rapport. A qualitative study with 15 GPs audio-recorded 25 to 30 consultations indicated that doctors may build up connection or rapport when seeing patients they were familiar with, and they may feel easy to talk to each other during the consultations [28]. In a study conducted in the Netherlands, GPs may also show concern when important life events occur for their patients, even when not specifically asked [29].

### Implication for research

This study compared the provision of patient-centred care between two groups of patients that GPs were familiar with or not during GP consultations in community health centres in China, which provided a basis for future research. Whether GPs provision of patient-centred care on the two groups, GP’s efforts toward ‘health knowledge’ and ‘patient knowledge’ on the familiar group, as well as efforts towards ‘chatting’ on the unfamiliar group, are enough could be further discussed. On the other hand, for the variable of familiarity, more research is needed to develop and validate the measurement of familiarity. In this study, we evaluate familiarity from GP’s perspective, therefore future studies could be conducted from both GP and patient perspective.

## Conclusion

Results of this study suggested that GP perceived doctor-patient familiarity may not be associated with GPs’ provision of patient-centred care during GP consultations in China’s context. This is remarkable as we expected that familiarity would enhance patient-centred care. Not unexpectedly, patients would show more towards health knowledge and ask more questions when GPs were not familiar with them during consultations. Further research is needed to confirm and expand on these findings.

## Data Availability

Data are available from the corresponding author on reasonable request.

## Abbreviations

GP: General practitioners
DOC: Davis Observation Code
CHCs: Community health centres
ANCOVAs: Multivariate analyses of covariances
